# Primary bladder diffuse large B-cell lymphoma causing unilateral ureterovesical junction obstruction: A case report

**DOI:** 10.1016/j.eucr.2026.103633

**Published:** 2026-10-01

**Authors:** Reza Kaffash Nayeri, Farshad Banouei, Maryam Abolhasani, Mostafa Rajabi

**Affiliations:** a Department of Urology, Hasheminejad Kidney Center, School of Medicine, Iran University of Medical Sciences, Tehran, Iran; b Department of Pathology, Hasheminejad Kidney Center, School of Medicine, Iran University of Medical Sciences, Tehran, Iran

## Abstract

Primary bladder diffuse large B-cell lymphoma is a rare extranodal malignancy that often mimics urothelial carcinoma. We report a 65-year-old male with flank pain and hematuria found to have a bladder mass extending to the ureterovesical junction with hydroureteronephrosis; urine cytology was repeatedly negative. Cystoscopic biopsy confirmed DLBCL, with no marrow involvement. Transurethral resection plus R-CHOP chemotherapy produced complete metabolic response on FDG-PET/CT, with stable residual uptake later deemed post-treatment change (Deauville 3). This case highlights ureteral obstruction as an unusual presentation and serial PET/CT as valuable for distinguishing residual lymphoma from fibrosis after therapy.

## 1 Introduction

Primary lymphoma of the urinary bladder is rare, with an estimated incidence of 0.3 cases per 1,000,000 individuals.^1^ Primary bladder diffuse large B-cell lymphoma (PB-DLBCL) accounts for only 0.2% of extranodal lymphomas.^2^ DLBCL and MALT/marginal zone lymphoma are the most common histological subtypes, and urinary symptoms such as hematuria, urgency, obstructive uropathy, or flank pain occur in the majority of patients.^3^ Clinical presentation is often nonspecific, mimicking urothelial carcinoma or recurrent urinary tract infection and posing a diagnostic challenge.^4^ Upper urinary tract obstruction is uncommon and occurs more often with advanced disease than with primary bladder lymphoma.^5^ However, when a lymphomatous mass involves the ureteral orifice, mechanical obstruction of the ureterovesical junction (UVJ) can cause hydronephrosis. Because imaging findings are not pathognomonic, definitive diagnosis relies on cystoscopy, histopathology, and immunohistochemistry, with tumor cells typically expressing CD20, CD79a, PAX-5, and BCL-6.^6^ We report a case of primary bladder lymphoma involving the ureteral orifice with consequent hydronephrosis, its diagnostic work-up, and its response to treatment.

Here, we present the case of a 65-year-old man who was found to have primary bladder diffuse large B-cell lymphoma causing unilateral ureterovesical junction obstruction and hydronephrosis, detailing his clinical presentation, diagnostic evaluation, and response to transurethral resection combined with systemic chemotherapy.

## 2 Case Presentation

A 65-year-old male presented with a 2-month history of mild right flank and lower abdominal pain and a single episode of gross hematuria. He was afebrile with stable vital signs, denied constitutional symptoms, chronic illness, or a significant family history, and had a 10-pack-year smoking history. Physical examination was unremarkable, without abdominal tenderness, organomegaly, or palpable masses. He reported no relevant psychosocial history and no known family history of malignancy or hereditary genetic syndromes. He had not received any prior treatment or intervention for these symptoms before this presentation.

A complete blood count showed a white blood cell count of 5800/μL with neutrophil predominance (73.6%) and relative lymphocytopenia (14.8%); serum creatinine was mildly elevated at 1.5 mg/dL, with other biochemical parameters normal. Urinalysis revealed marked microscopic hematuria, while urine cytology was negative for malignancy on three separate occasions.

Renal and bladder ultrasonography showed mild right hydroureteronephrosis and focal right bladder-wall thickening (∼3 cm). Contrast-enhanced CT of the abdomen and pelvis confirmed a mass-like, heterogeneously enhancing thickening of the posterolateral bladder wall extending to the right UVJ, distal right ureter, and right seminal vesicle, with mild right hydroureteronephrosis (Fig. 1). Perivesical (up to 8 mm) and right iliac chain (largest 13 mm) lymphadenopathy were noted, together with tumoral extension along the right pelvic sidewall involving the obturator muscles and internal iliac vessels. Chest CT showed no mediastinal lymphadenopathy or pulmonary involvement.

**Fig. 1:**
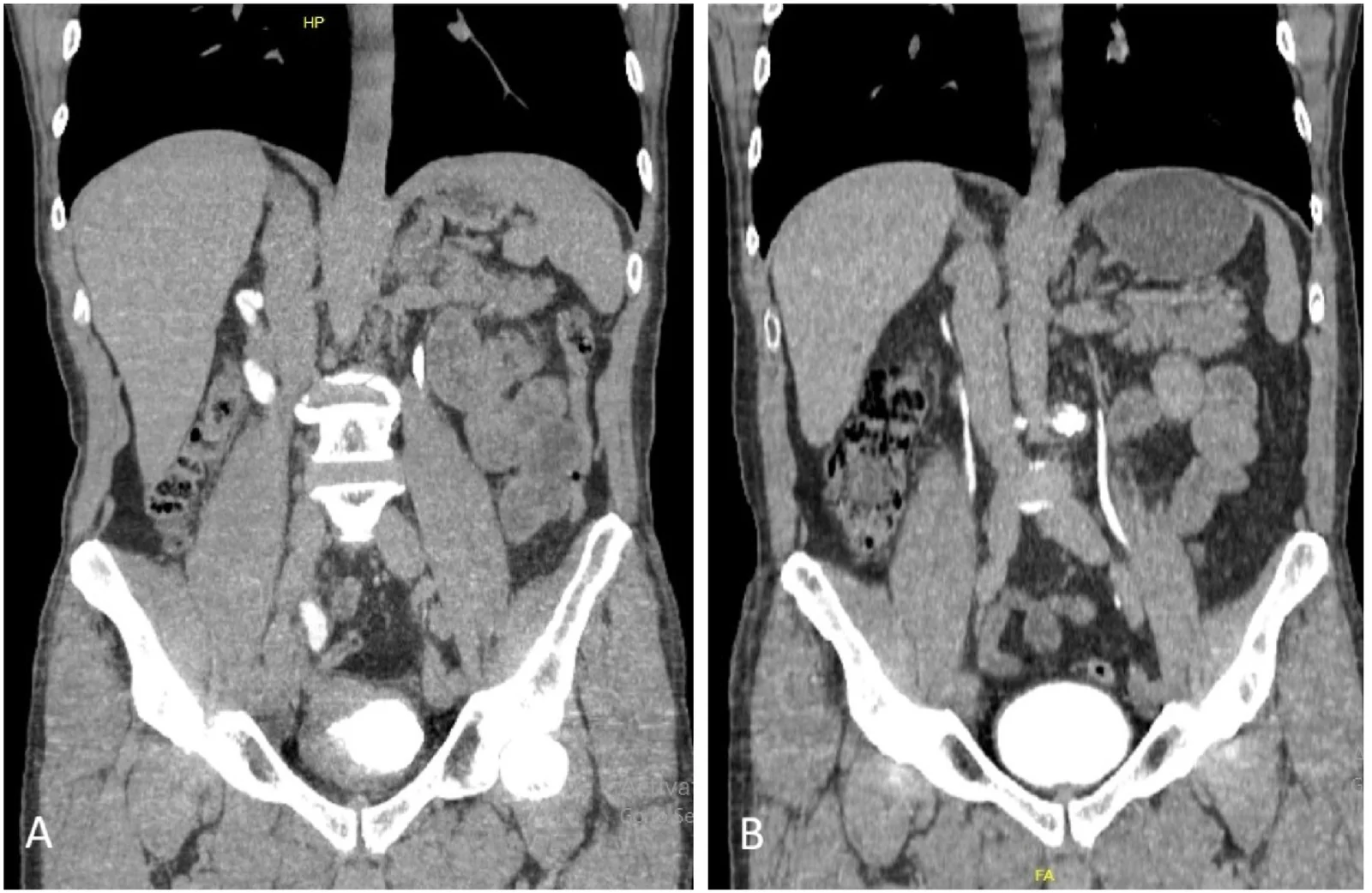
A. Coronal contrast-enhanced CT images showing right hydroureteronephrosis secondary to bladder mass involvement of the right ureterovesical junction. B. Resolution of hydronephrosis after complete treatment of the mass and smoothing of the bladder wall.

Timeline: The patient's course is summarized chronologically as follows. At presentation, a two-month history of right flank and lower abdominal pain with gross hematuria prompted evaluation; ultrasonography and contrast-enhanced CT demonstrated a bladder mass with right hydroureteronephrosis, and urine cytology was negative on three separate occasions. Cystoscopy with biopsy subsequently established the diagnosis of primary diffuse large B-cell lymphoma of the bladder, and staging bone marrow aspiration and trephine biopsy excluded systemic marrow involvement. The patient then underwent transurethral resection of the bladder tumor (TURBT), with placement of a right ureteral double-J stent to relieve the obstruction, followed by systemic chemotherapy. The stent was removed after completion of treatment, once the hydronephrosis had resolved. FDG-PET/CT performed after completion of treatment showed complete metabolic response. Approximately four months later, follow-up FDG-PET/CT demonstrated stable, faint residual uptake near the UVJ (Deauville score 3, interpreted as a healed lesion) together with an incidental focus of prostatic uptake.

Cystoscopy revealed a large, bulky mass occupying the posterolateral bladder wall that completely obscured the right ureteral orifice, mechanically accounting for the hydroureteronephrosis. Biopsy confirmed diffuse large B-cell lymphoma of the bladder. Histopathological examination showed atypical lymphoid cells beneath normal urothelium, with tumor cells positive for CD20 confirming B-cell origin, 40% Ki-67 proliferation index, and CD10 positivity confirming a germinal-center subtype (Fig. 2).

**Fig. 2:**
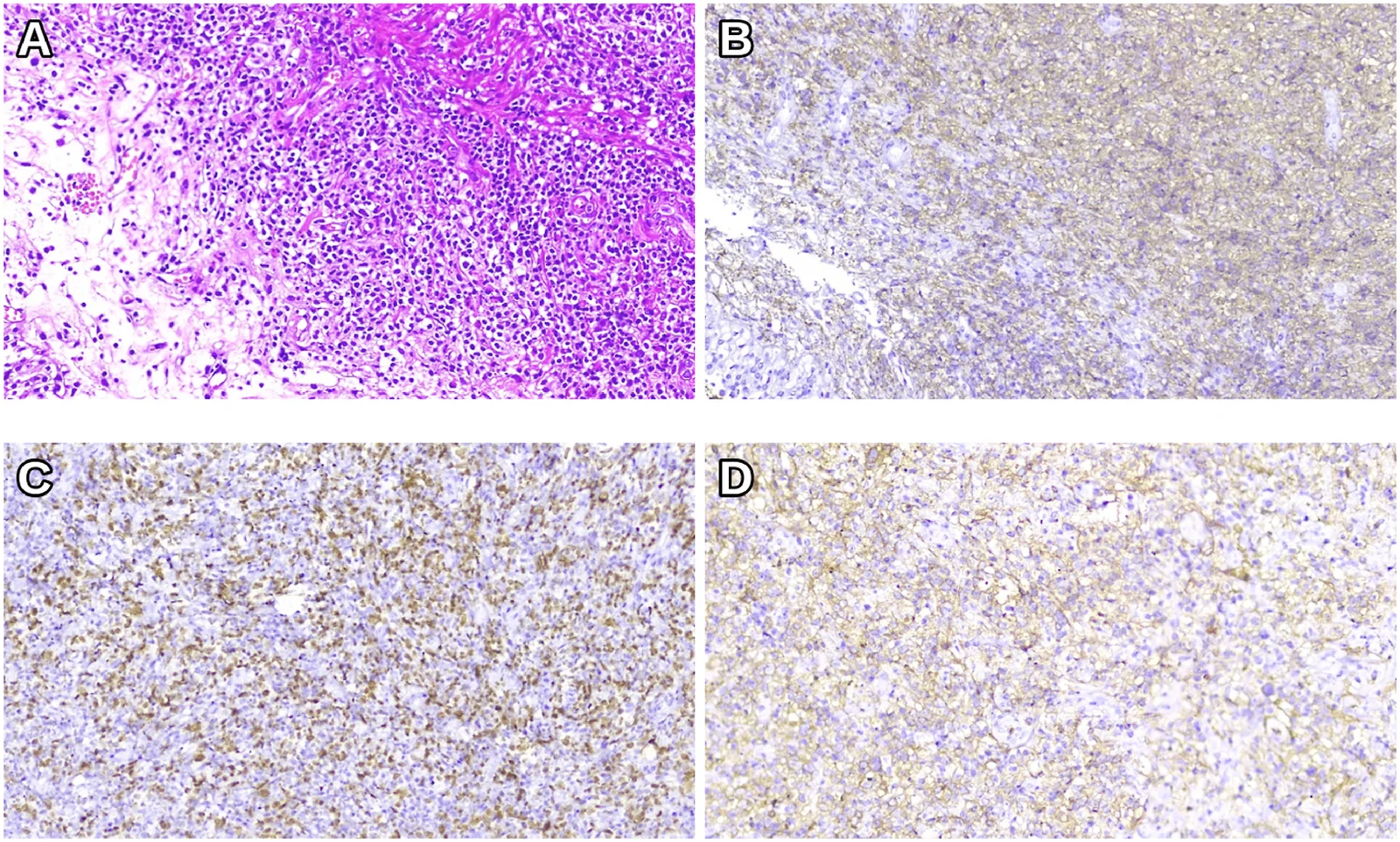
(A) The H&E stained slide showing atypical lymphoid cells beneath normal urothelium (x20 objective). (B) Tumor cells positive for CD20 by staining, confirming B-cell origin (x20 objective). (C) 40% of tumor cells showed positivity for Ki-67 by immunohistochemistry (x20 objective). (D) Atypical lymphoid cells positive for CD10 by immunohistochemistry, confirming the center subtype (x20 objective).

Given the rarity and nonspecific presentation of this entity, urothelial carcinoma, cystitis glandularis, and recurrent urinary tract infection were the principal alternative diagnoses considered before biopsy; the rarity of primary bladder lymphoma and the consequent lack of a standardized diagnostic pathway also posed a diagnostic challenge, and histopathological confirmation with immunohistochemistry was required to establish the diagnosis with certainty. Following staging that excluded systemic and marrow involvement (Ann Arbor stage IIE disease, reflecting the perivesical and right iliac chain lymphadenopathy noted on imaging), the prognosis was considered favorable, consistent with the generally good outcomes reported for localized primary bladder DLBCL treated with combined-modality therapy.

Staging bone marrow aspiration and trephine biopsy demonstrated an almost normocellular marrow (25–40% cellularity) with a myeloid-to-erythroid ratio of 3.8:1, 1% myeloblasts, and no significant dysplasia; lymphoid forms were predominantly small mature cells without atypia. Immunohistochemistry (CD5, CD19, CD20, PAX5, CD79α, CD138) showed only rare, scattered positive cells, with no lymphoid aggregates, thereby excluding marrow involvement by lymphoma. Concordant flow cytometry immunophenotyping of the aspirate identified a lymphoid population comprising 10% of analyzed cells, predominantly mature T cells, with a small (4%) polytypic mature B-cell subset showing no surface light-chain restriction, 2% hematogones, and 1% myeloblasts; no CD45-negative or immunophenotypically abnormal population was identified, confirming the absence of marrow infiltration.

The patient underwent transurethral resection of the bladder tumor (TURBT), with placement of a right ureteral double-J stent to relieve the obstruction, followed by systemic chemotherapy with R-CHOP (rituximab, cyclophosphamide, doxorubicin, vincristine, and prednisone), administered for six cycles at 21-day intervals. The stent was removed after completion of treatment, once the hydronephrosis had resolved. FDG-PET/CT performed after treatment completion (injected dose 9.6 mCi ^18^F-FDG) showed no metabolically active tumoral lesion throughout the body, consistent with complete metabolic response.

On follow-up FDG-PET/CT performed approximately four months later to evaluate possible recurrence, faint, fairly uniform FDG uptake (SUVmax 1.8) with soft-tissue stranding persisted adjacent to the distal right ureter just proximal to the UVJ (Deauville score 3), stable compared with the prior study and interpreted as a healed lesion consistent with sustained complete metabolic remission (Fig. 3). No other metabolically active disease was identified. An incidental focus of increased uptake (SUVmax 8) was noted in the right peripheral zone of the prostate gland, for which serum PSA measurement and sonographic evaluation were recommended. On subsequent evaluation, PSA was normal and sonography was benign, excluding prostatic malignancy.

**Fig. 3:**
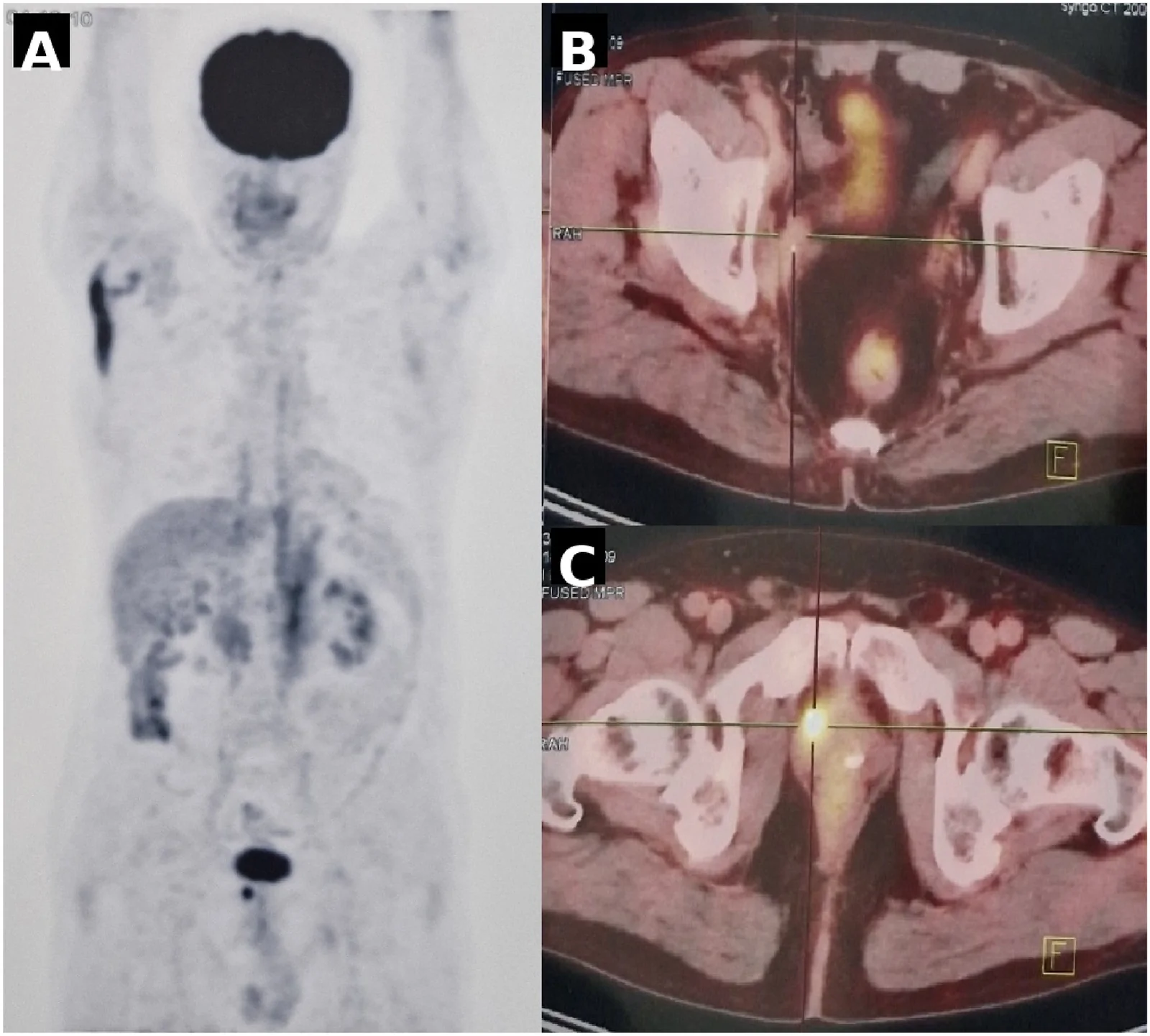
Follow-up FDG-PET/CT. **A.** Coronal maximum-intensity-projection image demonstrating faint residual uptake adjacent to the distal right ureter/UVJ (healed lesion, Deauville score 3). **B.** Fused axial image at the level of the distal right ureter/UVJ showing the same faint residual uptake. **C.** Fused axial image showing the incidental focus of uptake in the right peripheral zone of the prostate gland.

## 3 Discussion

PB-DLBCL is thought to arise from acquired lymphoid tissue induced by chronic bladder inflammation, since the bladder normally lacks native lymphoid tissue.^1^^,^^2^ It behaves far more aggressively than the more common indolent MALT/marginal zone subtype at this site, and evidence is limited mainly to case reports. This case highlights two underreported features: mechanical UVJ obstruction as the presenting complication and serial FDG-PET/CT, interpreted using the Deauville criteria, to confirm durable remission.

Diagnosis is often delayed because PB-DLBCL mimics urothelial carcinoma, cystitis glandularis, or recurrent urinary tract infection on imaging and symptoms.^4^ A useful clue is negative urine cytology, seen on three occasions in this patient, since lymphoma infiltrates beneath an intact urothelium and sheds few malignant cells, unlike urothelial carcinoma. Cystoscopic biopsy with immunohistochemistry (CD20, CD79a, PAX-5, BCL-6) remains the diagnostic standard.^6^^,^^7^ Marrow evaluation is needed to exclude secondary, systemic involvement before disease is labeled primary.

Upper urinary tract obstruction from primary bladder lymphoma is uncommon and typically bilateral or associated with advanced disease.^5^ Unilateral hydronephrosis from direct tumor encasement of a single ureteral orifice, as in this case, is a less frequently described mechanism and worth considering in older adults with a bladder mass and unilateral hydroureteronephrosis, even though urothelial carcinoma remains statistically more likely.^3^

No standardized treatment protocol exists given the disease's rarity, with reported approaches ranging from transurethral resection alone to radical cystectomy, chemotherapy, or combinations thereof.^7^ Given the aggressive histology, anthracycline-based immunochemotherapy (R-CHOP: rituximab, cyclophosphamide, doxorubicin, vincristine, and prednisone), as administered in this patient for six cycles at 21-day intervals, is generally favored, with transurethral resection used mainly for tissue diagnosis and local debulking rather than definitive therapy — the approach taken here, which achieved a complete metabolic response.

Response was assessed with the Deauville five-point scale used in the Lugano classification.^8^ The faint, stable uptake (SUVmax 1.8, Deauville score 3) persisting near the UVJ on follow-up imaging could have reflected either post-treatment fibrosis or residual disease; it was stability across sequential scans, rather than a single study, that supported classifying it as a healed lesion. This underscores the value of serial over single-time-point metabolic imaging. The incidental prostatic uptake (SUVmax 8) is a reminder that whole-body PET surveillance can detect unrelated findings warranting separate work-up rather than being conflated with lymphoma recurrence.

This report is limited by its single-patient design and relatively short follow-up, precluding conclusions about long-term durability of remission. Nonetheless, it reinforces that primary bladder lymphoma should remain in the differential of a bladder mass with unilateral ureteral obstruction, that tissue diagnosis is indispensable given nonspecific imaging and cytology, and that sequential FDG-PET/CT helps distinguish residual disease from post-treatment change.

Primary bladder lymphoma is rare and can mimic urothelial malignancy, occasionally causing ureteral obstruction through UVJ involvement, as in this case. Cystoscopic biopsy and immunohistochemistry remain essential for diagnosis, since imaging and urine cytology alone are often nonspecific. Sequential FDG-PET/CT confirmed a durable complete metabolic response to TURBT and chemotherapy, underscoring the value of metabolic imaging in monitoring remission, while the incidental prostatic uptake — subsequently confirmed benign on PSA and sonographic evaluation — illustrates the need for separate clinical follow-up of unrelated findings.

## CRediT authorship contribution statement

**Reza Kaffash Nayeri:** Conceptualization. **Farshad Banouei:** Writing – review & editing, Writing – original draft, Supervision. **Maryam Abolhasani:** Visualization. **Mostafa Rajabi:** Data curation.

## Informed consent

Written informed consent was obtained from the patient for publication of this case report and any accompanying images.

## Data availability

Data are available from the corresponding author on reasonable request.

## Ethics approval and consent to participate

This case report was reviewed and approved by the Institutional Review Board (IRB) of Hasheminejad Kidney Center, Iran University of Medical Sciences.

## Consent for publication

Written informed consent was obtained from the patient for the publication of this case report and any accompanying images and associated clinical details.

## Availability of data and materials

The datasets generated and/or analyzed during the current case report are available from the corresponding author on reasonable request.

## Declaration of generative AI and AI-assisted technologies in the manuscript preparation process

During the preparation of this work, the author(s) used Claude (Anthropic, Claude Sonnet 5) in order to reduce word count to meet journal requirements and to revise the language of the Background, Case Presentation, and Discussion sections for clarity and readability. After using this tool, the author(s) reviewed and edited the content as needed and take full responsibility for the content of the published article. No scientific content, data, interpretation, or citation was added, altered, or removed beyond the original manuscript's meaning, and the AI tool had no influence on the study's conclusions, data analysis, or scientific interpretation.

## Funding

None declared.

## Declaration of competing interests

The authors declare that they have no known competing financial interests or personal relationships that could have appeared to influence the work reported in this paper.
